# Options for first line therapy of Hodgkin lymphoma

**DOI:** 10.1002/hon.2597

**Published:** 2019-06-12

**Authors:** Jemma Longley, Peter W.M. Johnson

**Affiliations:** ^1^ Cancer Research UK Centre University of Southampton UK

## 1 INTRODUCTION

Treatment for Hodgkin lymphoma (HL) has evolved over the last 50 years from extended field radiotherapy to the use of multimodality regimens tailored to individual patients' disease characteristics, with the recent addition of antibody‐drug conjugate therapies and immune checkpoint inhibitors. This progress has translated into 10‐year survival rates above 80% with current standard treatment approaches, although long‐term follow‐up of these survivors shows that a minority experience significant morbidity and mortality as a result of their treatment, such as second malignancies and cardiovascular disease.^1^ This has led to a more individualised approach to treatment, using risk‐ and response‐adapted strategies, with the aim of de‐escalating treatment and minimising toxicity without compromising survival in patients whose disease can be cured by less toxic regimens, while intensifying treatment in patients with high risk disease who stand to gain the most benefit. The general improvement in outcomes has not been matched in the older population, where there is as yet no standard treatment approach, and we lack clinical trial data to support decision making in patients more than the age of 60.

## 2 RISK‐ AND RESPONSE‐ADAPTED THERAPY

Advanced stage HL has been recently treated with either ABVD or BEACOPP chemotherapy regimens, mainly according to national preferences. Excellent disease control rates have been demonstrated with the more intensive BEACOPP regimens, but at the cost of more acute toxicity and long‐term morbidity.^2^ The risk stratification of patients with advanced stage disease currently uses the International Prognostic Score (IPS) at diagnosis to inform initial treatment decisions, although it does not clearly identify any group with a very poor outlook. Attempts to modify IPS using gene expression profiles have not proven reproducible, and deciding which patients should initially receive more intensive chemotherapy regimens remains challenging. Prospective studies incorporating novel biomarkers or baseline imaging features, such as the metabolic tumour volume, may help to develop a robust prediction model and aid clinical decision making.

**Figure 1:**
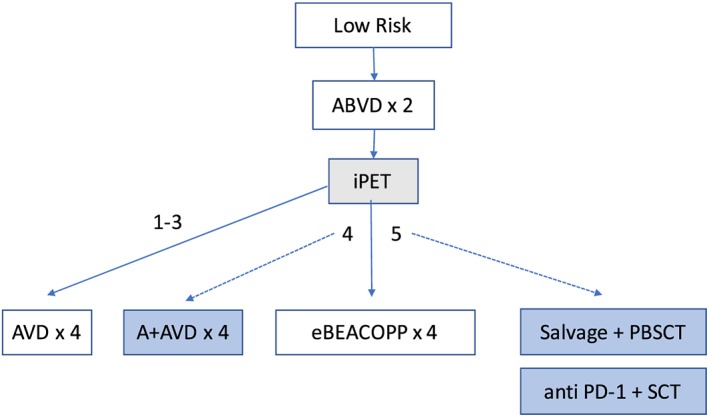
Potential treatment algorithm for low‐risk advanced stage Hodgkin lymphoma (HL) (experimental approaches are shown in shaded boxes)

The use of 2‐(^18^F)‐fluoro‐2‐deoxy‐D‐glucose (FDG) positron emission tomography (PET) combined with CT has allowed the metabolic characterisation of disease sites. This was validated in a retrospective study where patients with a negative interim PET scan following two cycles of ABVD had a 3‐year progression free survival rate (PFS) of 95%, while those with a positive interim PET had a 3‐year PFS of only 13%, independent of IPS at diagnosis.^3^ In order to improve the reproducibility of PET scan scoring, the Deauville 5‐point scoring system has been developed (Table 1). Its use was investigated prospectively by both the LYSA AHL2011 and RATHL randomised phase III trials in advanced stage disease (Stage IIB‐IV).^4^ Patients enrolled in Response‐Adapted Therapy in advanced Hodgkin's Lymphoma (RATHL) received two cycles of ABVD and following a negative interim PET (iPET, defined as Deauville score 1‐3) were randomised to receive a further four cycles of either ABVD or AVD.^5^ The omission of bleomycin in cycles 3 to 6 resulted in similar 3‐year PFS and overall survival (OS) rates compared with those patients receiving a total of six cycles of ABVD, with reduced pulmonary toxicity (3‐year PFS 84% versus 86% and OS 98% versus 97%, respectively). This led to the conclusion that bleomycin can be safely excluded from treatment in patients with early complete metabolic response. In the same trial, patients with a positive iPET were escalated to BEACOPP regimens, resulting in a 3‐year PFS of 68%, which compared favourably to outcomes in studies in which patients continued with ABVD despite a positive iPET. The LYSA AHL2011 trial started with two cycles of the more intensive escBEACOPP regimen. Five‐year PFS and OS rates did not significantly differ between patients treated with a standard approach, who received six cycles of escBEACOPP, compared with those in the experimental arm where the 84% with a negative iPET then received four cycles of ABVD. The overall PFS by intention to treat was 86.2% versus 85.7%, respectively, and among those with a negative iPET, 88.4% versus 89.4%.^6^ The German Hodgkin Study Group (GHSG) established that the number of escBEACOPP cycles could be reduced from six to four following a negative iPET (here defined as Deauville score 1‐2) without compromising disease control. Patients receiving a total of four cycles of escBEACOPP showed a small improvement in 5‐year PFS compared with six or eight cycles (92.2% vs. 90.8%) and 5‐year OS (97.7 vs. 95.4%).^2^ The negative predictive value of iPET is demonstrably higher following more intensive regimens, especially in patients with high risk disease, leading to the conclusion that patients with Stage IV disease and/or high IPS may be best managed initially with two cycles of escBEACOPP. For patients with a low IPS who have a high chance of cure with ABVD, starting with this regimen reduces the acute haematological toxicity compared with escBEACOPP, but still produces high rates of disease control. In both scenarios, de‐escalation of treatment following a negative iPET is justified, based on recent clinical trial data (see Figure 1).

**Table 1:** The Deauville 5‐point scoring system for response assessment

1	No uptake
2	Uptake ≤ mediastinum
3	Uptake > mediastinum but ≤ liver
4	Moderately increased uptake > liver
5	Markedly increased uptake > liver and/or new lesions related to lymphoma

Despite escalation to BEACOPP regimens in the RATHL trial, among patients with an iPET score of 5 the treatment failed in 20 of 37 (54%) cases, indicating that this small group of patients requires a different approach. The Italian HD0801 study investigated the use of high‐dose ifosfomide‐based salvage chemotherapy with autologous or allogenic stem cell transplant following an iPET score of 3 to 5, resulting in a 2‐year PFS of 75%; similar to the 81% seen in patients who were iPET negative.^7^ The early use of high‐dose intensification in the small patient population genuinely refractory to primary chemotherapy may offer advantages for long‐term disease control.

The omission of consolidation radiotherapy (RT), following a negative iPET in the RATHL trial, resulted in only 6.5% of patients overall receiving RT, without apparent loss of disease control.^5^ SImilarly, the GHSG HD15 trial found that the omission of consolidation RT in patients with a negative iPET scan and residual masses of less than 2.5 cm did not compromise overall treatment efficacy.^8^ This was confirmed in a randomised trial by the GITL/FIL group, who assigned patients with a large nodal mass at diagnosis (greater than 5 cm) to radiotherapy versus no further treatment in those with complete metabolic remission at iPET and the end of treatment.^9^ There was no significant difference in 3‐year PFS between the two groups (97% vs. 93% *P* = 0.29) leading to the conclusion that RT can be safely omitted in patients with a negative iPET, even if they present with bulky disease. The use of RT for patients with a persistent single site FDG‐avid focus may avoid the need for more intensive chemotherapy regimes, but this approach has not been validated in prospective studies.

The treatment strategy for early stage disease has increasingly emphasised reducing long‐term toxicity without compromising the excellent OS rates of over 95% at 10 years. Recent research has investigated the omission of radiotherapy for patients with a negative iPET. In the UK NCRI RAPID trial, patients with non‐bulky Stage IA and IIA disease and a negative iPET defined as Deauville score 1 to 2, following three cycles of ABVD (75% of cases), were randomised to receive no further treatment or involved field radiotherapy.^10^ There was a small but significant increase in recurrence rate in patients who did not receive radiotherapy, with a 3‐year PFS of 94.6% in the radiotherapy group and 90.8% in the group who received only three ABVD, without a worsening of 3‐year OS. A trial conducted by the EORTC, LYSA, and FIL groups (HD10) showed similar effects. In iPET‐negative patients after two ABVD, the 5‐year PFS rates in the favourable group who received either one ABVD plus Involved Node Radiotherapy or two further ABVD were 99.0% versus 87.1%, while in the unfavourable group who received either two ABVD plus INRT or four more ABVD, the 5‐year PFS figures were closer, at 92.1% versus 89.6%.^11^ The overall survival figures in both studies were very high, which argues for an individualised approach to treatment, balancing the small increase in the risk of recurrence against the potential morbidity of local radiotherapy. This will depend upon the particular sites of involvement and other factors such as gender and co‐morbidity. For the future, the baseline evaluation of metabolic tumour volume and total lesional glycolysis may be helpful in estimating the risk from a radiation‐free approach.^12^

## 3 INCORPORATION OF BRENTUXIMAB VEDOTIN

Brentuximab Vedotin (BV) is an anti‐CD30 monoclonal antibody linked to an anti‐microtubule agent that is internalised by the target cell and cleaved, resulting in cell cycle arrest. Early clinical trials testing its combination with chemotherapy regimens containing bleomycin caused severe pulmonary toxicity, but it was found to be safe in combination with AVD, leading to the development of the ECHELON‐1 trial, comparing six cycles of AVD with BV against ABVD in patients with stage III/IV disease.^13^ This study was not blinded and had modified PFS as its primary endpoint, including as an event the administration of further therapy at the end of treatment for patients with a Deauville Score of 3 or above. The 2‐year modified PFS results showed a 4.9% difference favouring the BV group, at the cost of increased grade 3 to 4 peripheral neuropathy and neutropenia, mitigated by the routine administration of growth factors. Interestingly, a subgroup analysis comparing outcomes by stage showed that benefit from the addition of BV was largely confined to those with stage IV disease, who had a statistically significant improvement of 6.7% in 2‐year PFS, compared with only 1.1% in patients at Stage III. The incorporation of BV into first line regimens might therefore benefit those patients who present with high‐risk disease, offering apparently better disease control than ABVD. The incorporation of BV into BEACOPP‐like regimens is the subject of the ongoing GHSG HD21 trial. BrECADD (BV, etoposide, cyclophosphamide, doxorubicin, dacarbazine, and dexamethasone) was selected for comparison with escBEACOPP following a phase II evaluation which showed a complete response rate of 88% in patients receiving 6 cycles, with no Grade 3 to 4 peripheral neuropathy.^14^

The use of BV combined with AVD in early stage unfavourable HL, followed by involved site radiotherapy (ISRT), did not appear to increase the rate of acute pulmonary toxicity while producing a high response rate in the Phase II study.^15^ Patients received four cycles of BV + AVD with iPET following cycles 2 and 4. Treatment was not modified based on imaging, and 90% patients achieved a negative iPET (Deauville score 1‐3) after two cycles. The 1 year PFS was 93.3% and no patient had grade ≥ 3 pneumonitis. Whether radiotherapy could be omitted following this highly active regimen, given the high proportion of patients with a negative iPET, remains an open question, but it may be an attractive option for patients with bulky disease (defined as greater than 10 cm); 85% of whom achieved a negative iPET after only two cycles of treatment.

In the older population, BV may offer an alternative for patients unable to receive anthracyclines or bleomycin because of cardiorespiratory co‐morbidity. It has been tested in combination with bendamustine or dacarbazine for patients with three or more co‐morbidities unable to receive conventional regimens,^16^ but there was a prohibitive rate of serious adverse events associated with BV plus bendamustine. By contrast, BV plus dacarbazine gave an overall response rate (ORR) of 100% with complete responses in 62%, and a more favourable toxicity profile.

Sequential therapy with two cycles of BV followed by six cycles of AVD was investigated in a Phase II trial among 48 patients, with a median age of 67 and performance status of 1.^17^ Patients whose disease responded to treatment received a further four cycles of BV monotherapy. The 2‐year PFS within the intention to treat population was 84%, with OS 93%, making this a potentially attractive option for fit older patients.

## 4 CHECKPOINT BLOCKING ANTIBODIES

Cytogenetic studies have shown an amplification in copy number of the programmed cell death ligand (PDL1 and PDL2) gene loci in the majority of HL cases, leading to the up‐regulation of *PDL1* and *PDL2* surface expression.^18^ Nivolumab is an antiprogrammed cell death (anti‐PD1) IgG4 monoclonal antibody, currently licenced for relapsed HL, which targets the *PD1* T‐cell surface receptor interaction. This disrupts the interaction between the malignant B‐cell population and the surrounding T‐cells, potentially depriving the malignant cells of growth signals, and disinhibiting a cytotoxic T‐cell response. In advanced stage disease, the Checkmate 205 Phase II trial investigated initial therapy with four doses of nivolumab monotherapy followed by six cycles of combination therapy with AVD.^19^ No treatment‐related deaths were reported, and no serious pulmonary toxicity. The regimen was well‐tolerated with an ORR of 84% at the end of treatment. Median time to respond was 2 months and at 9 months, the PFS was 94%.

Given the impressive response rates in heavily pretreated patients, anti‐PD1 antibodies may prove useful in primary refractory disease or for patients with a positive iPET, where alternative treatment strategies are still needed. Within the first line setting, there is a need to better understand the biology of the HL microenvironment by developing biomarkers to establish which patients may benefit most from the addition of these antibodies to what are already very effective frontline chemotherapy regimens.

## 5 CONCLUSIONS

**Figure 2:**
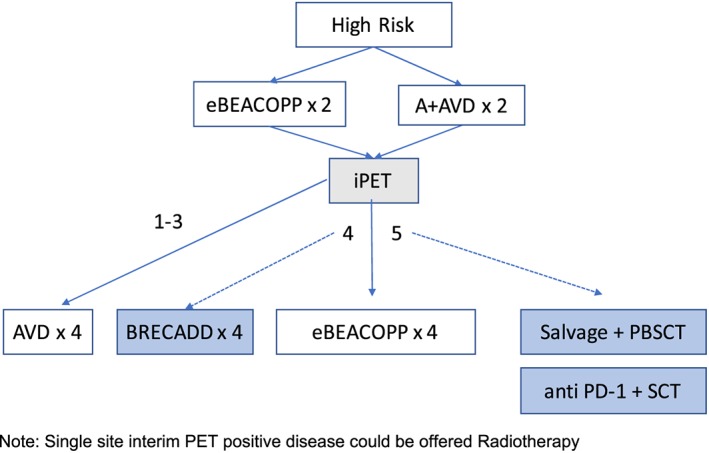
Potential treatment algorithm for high‐risk advanced stage Hodgkin lymphoma (HL) (experimental approaches are shown in shaded boxes). Note: Single site interim PET positive disease could be offered radiotherapy

The treatment of HL in the modern era continues to evolve, with the incorporation of new targeted therapies that offer additions or alternatives to conventional chemotherapy regimens. Their optimal use in different patient populations, either as monotherapy or in combinations remains a challenge, and there is a continued need for predictive biomarkers to select those patients most likely to benefit. Figures 1 and 2 summarise our current thinking on the standard and experimental approaches to treatment for advanced disease. The use of PET response‐adapted therapy in this context is an attractive one, and the role of the new treatments in the frontline setting may be realised in the context of high‐risk or poorly‐responding disease or in older patient groups.
